# Supplementary material for: Genetic diversity and population history of eight Italian beef cattle breeds using measures of autozygosity
Source: PLoS One. 2021 Oct 25;16(10):e0248087. doi: 10.1371/journal.pone.0248087 (PMC8544844; doi:10.1371/journal.pone.0248087)
Supplement: S1 Table — (DOCX) [file pone.0248087.s001.docx]

| **BTA** | **CAL** | **CHA** | **LIM** | **MUP** | **PON** | **SAB** | **SAM** | **SAR** |
| --- | --- | --- | --- | --- | --- | --- | --- | --- |
| 1 | 2,873 | 2,956 | 3,312 | 2,543 | 599 | 3,010 | 1,439 | 2,821 |
| 2 | 2,180 | 2,838 | 2,969 | 2,679 | 588 | 2,676 | 1,355 | 2,712 |
| 3 | 1,907 | 2,266 | 2,353 | 2,284 | 491 | 2,245 | 1,084 | 2,286 |
| 4 | 2,130 | 2,647 | 2,731 | 2,311 | 477 | 2,475 | 1,190 | 2,612 |
| 5 | 2,274 | 2,656 | 2,649 | 2,108 | 534 | 2,393 | 1,092 | 2,239 |
| 6 | 2,483 | 2,583 | 2,726 | 2,323 | 472 | 2,542 | 1,304 | 2,440 |
| 7 | 1,971 | 2,436 | 2,556 | 2,038 | 473 | 2,319 | 935 | 2,300 |
| 8 | 2,270 | 2,719 | 3,003 | 2,223 | 528 | 2,699 | 1,165 | 2,641 |
| 9 | 1,864 | 2,102 | 2,236 | 1,967 | 435 | 2,004 | 1,048 | 2,098 |
| 10 | 1,885 | 2,178 | 2,132 | 1,965 | 432 | 1,943 | 959 | 2,187 |
| 11 | 1,856 | 2,042 | 2,185 | 1,882 | 405 | 2,047 | 977 | 2,077 |
| 12 | 1,483 | 1,842 | 1,937 | 1,847 | 357 | 1,706 | 796 | 1,720 |
| 13 | 1,963 | 2,366 | 2,402 | 2,024 | 483 | 2,197 | 1,026 | 2,265 |
| 14 | 1,791 | 2,077 | 2,310 | 1,712 | 405 | 2,059 | 933 | 2,063 |
| 15 | 1,263 | 1,523 | 1,694 | 1,388 | 299 | 1,500 | 712 | 1,521 |
| 16 | 1,207 | 1,578 | 1,633 | 1,355 | 303 | 1,488 | 699 | 1,600 |
| 17 | 1,212 | 1,344 | 1,408 | 1,138 | 338 | 1,299 | 640 | 1,261 |
| 18 | 1,094 | 1,169 | 1,201 | 1,127 | 225 | 1,043 | 534 | 1,049 |
| 19 | 905 | 1,095 | 1,060 | 1,080 | 225 | 1,009 | 498 | 1,053 |
| 20 | 865 | 1,105 | 1,377 | 931 | 236 | 1,036 | 550 | 1,116 |
| 21 | 1,184 | 1,496 | 1,632 | 1,263 | 323 | 1,574 | 711 | 1,534 |
| 22 | 908 | 1,072 | 1,145 | 850 | 202 | 1,034 | 477 | 1,028 |
| 23 | 824 | 777 | 902 | 938 | 178 | 782 | 368 | 736 |
| 24 | 997 | 1,102 | 1,220 | 945 | 237 | 1,121 | 586 | 1,088 |
| 25 | 588 | 624 | 700 | 553 | 119 | 549 | 266 | 630 |
| 26 | 838 | 841 | 953 | 896 | 186 | 785 | 385 | 877 |
| 27 | 536 | 691 | 751 | 696 | 124 | 633 | 304 | 651 |
| 28 | 732 | 758 | 884 | 776 | 182 | 743 | 315 | 792 |
| 29 | 790 | 929 | 949 | 906 | 188 | 865 | 410 | 942 |

**S1 Table**
