# Supplementary material for: Genetic diversity and population history of eight Italian beef cattle breeds using measures of autozygosity
Source: PLoS One. 2021 Oct 25;16(10):e0248087. doi: 10.1371/journal.pone.0248087 (PMC8544844; doi:10.1371/journal.pone.0248087)
Supplement: S2 Table — (DOCX) [file pone.0248087.s002.docx]

**S2 Table**

| **Breed** | **CHR** | **Start SNP** | **End SNP** | **N. SNPs** | **from** | **to** | **Length (Mbp)** |
| --- | --- | --- | --- | --- | --- | --- | --- |
| CAL | 3 | RNF11 | BovineHD0300027507 | 3 | 95,601,697 | 95,753,782 | 0.15 |
|  | 6 | BovineHD0600010715 | Hapmap26233-BTA-75846 | 66 | 38,698,886 | 40,183,935 | 1.49 |
|  | 6 | BovineHD0600020167 | BovineHD0600034501 | 3 | 72,625,498 | 72,991,427 | 0.37 |
|  | 7 | BovineHD0700014803 | BovineHD0700015160 | 10 | 50,751,321 | 52,460,183 | 1.71 |
|  | 13 | Hapmap47850-BTA-118310 | BovineHD1300017027 | 9 | 58,904,174 | 59,467,689 | 0.56 |
|  | 13 | BovineHD4100010307 | BTA-112783-no-rs | 12 | 63,242,901 | 63,715,266 | 0.47 |
|  | 13 | ARS-BFGL-NGS-17925 | BovineHD1300018419 | 9 | 63,907,611 | 64,683,488 | 0.78 |
|  | 13 | BovineHD1300018513 | BovineHD1300018659 | 8 | 65,161251 | 65,721,796 | 0.56 |
|  | 13 | BovineHD1300018795 | BovineHD1300018836 | 4 | 66,230,066 | 66,402,921 | 0.17 |
|  | 14 | BovineHD1400013371 | BovineHD1400013441 | 3 | 47,279,060 | 47,535,651 | 0.26 |
|  | 16 | BovineHD1600000011 | BovineHD1600000286 | 17 | 99,900 | 1,163,809 | 1.06 |
| CHA | 5 | BovineHD0500016070 | BovineHD0500016088 | 18 | 56,625,841 | 56,716,286 | 0.09 |
|  | 5 | BovineHD0500016090 | BovineHD0500016469 | 52 | 56,722,571 | 58,464,570 | 1.74 |
|  | 13 | ARS-BFGL-NGS-4243 | BovineHD1300003267 | 5 | 10,966,294 | 11,842,431 | 0.88 |
|  | 21 | BovineHD2100000012 | BovineHD2100000219 | 18 | 83,766 | 1,786,020 | 1.70 |
|  | 21 | BovineHD2100021044 | BovineHD2100000258 | 2 | 1,854,171 | 2,085,345 | 0.23 |
| LIM | 2 | ARS-BFGL-NGS-21306 | BTB-01111412 | 35 | 5,305,197 | 7,958,492 | 2.65 |
|  | 13 | BovineHD1300003108 | BovineHD1300003267 | 4 | 11,397,610 | 11,842,431 | 0.44 |
|  | 14 | BovineHD1400007190 | Hapmap40958-BTA-34312 | 29 | 24,754,549 | 26,450,034 | 1.70 |
|  | 21 | BovineHD2100000012 | BovineHD2100000320 | 25 | 83,766 | 2,467,774 | 2.38 |
| MUP | 5 | 5_74257621 | BovineHD0500021258 | 4 | 74,257,621 | 74,762,971 | 0.51 |
|  | 5 | 5_74951342 | 5_75130860 | 19 | 74,951,342 | 75,130,860 | 0.18 |
|  | 7 | BovineHD0700014803 | BovineHD0700015160 | 11 | 50,751,321 | 52,460,183 | 1.71 |
|  | 7 | BovineHD0700015212 | BovineHD0700015266 | 1 | 52,728,318 | 53,007,998 | 0.28 |
|  | 11 | BovineHD1100017061 | Hapmap41117-BTA-99065 | 9 | 59,628,354 | 60,548,547 | 0.92 |
|  | 11 | Hapmap48973-BTA-99093 | BovineHD1100017358 | 3 | 60,706,511 | 60,928,551 | 0.22 |
|  | 12 | BovineHD1200027369 | BovineHD1200013148 | 2 | 47,684,978 | 47,833,336 | 0.15 |
|  | 21 | BovineHD2100000012 | BovineHD2100000283 | 23 | 83,766 | 2,256,102 | 2.17 |
| PON | 5 | BovineHD0500021258 | 5_75130860 | 20 | 74,762,971 | 75,130,860 | 0.37 |
|  | 13 | BovineHD1300015452 | BovineHD1300015609 | 5 | 54,453,339 | 54,979,764 | 0.53 |
|  | 14 | BovineHD1400013371 | BovineHD1400013483 | 5 | 47,279,060 | 47,665,095 | 0.39 |
|  | 21 | BovineHD2100000012 | BovineHD2100000219 | 18 | 83,766 | 1,786,020 | 1.70 |
| SAB | 21 | BovineHD2100000012 | BovineHD2100000219 | 17 | 83,766 | 1,786,020 | 1.70 |
| SAM | 21 | BovineHD2100000012 | BovineHD2100000258 | 20 | 83,766 | 2,085,345 | 2.00 |
| SAR | 21 | BovineHD2100000012 | BovineHD2100000258 | 20 | 83,766 | 2,085,345 | 2.00 |

Assembly Bos_taurus_UMD_3.1
