# Supplementary material for: Genetic diversity and population history of eight Italian beef cattle breeds using measures of autozygosity
Source: PLoS One. 2021 Oct 25;16(10):e0248087. doi: 10.1371/journal.pone.0248087 (PMC8544844; doi:10.1371/journal.pone.0248087)
Supplement: S3 Table — (DOCX) [file pone.0248087.s003.docx]

**S3 Table**

|  | **CHR** | **Start_SNP** | **End_SNP** | **Genes in ROH** |
| --- | --- | --- | --- | --- |
| **CAL** | 6 | BovineHD0600010715 | Hapmap26233-BTA-75846 | *SLIT2, MIR218-1* |
|  | 16 | BovineHD1600000011 | BovineHD1600000286 | *OR5L1, TRNAR-UCU, TMEM183A,*  *PPFIA4, MYOG, ADORA1, MYBPH,*  *CHI3L1, CHIT1, BTG2, FMOD* |
| **CHA** | 5 | BovineHD0500016070 | BovineHD0500016088 | *HSD17B6, TRNAG-CCC, PRIM1* |
|  | 5 | BovineHD0500016090 | BovineHD0500016469 | *PRIM1, PTGES3, NACA, ATP5F1B,*  *MIR677, BAZ2A, RBMS2, GLS2, SPRYD4, MIP, TIMELESS, APOF, ApoN, STAT2, IL23A, MIR2432, PAN2, CNPY2, CS, COQ10A, ANKRD52, MIR2433, SLC39A5, NABP2, RNF41, TRNAS-CGA, SMARCC2, MYL6, MYL6B, ESYT1, ZC3H10, PA2G4, ERBB3, RPS26, IKZF4, SUOX, RAB5B, CDK2, PMEL, DGKA, PYM1, MMP19, DNAJC14, ORMDL2, SARNP, GDF11, CD63, RDH5, BLOC1S1, ITGA7, METTL7B, OR10P1, OR6C4, OR2AP1, OR6C2, OR6C68, OR6C68* |
| **LIM** | 2 | ARS-BFGL-NGS-21306 | BTB-01111412 | *CYP27C1, BIN1, MIR2350*  *NAB1, NEMP2, MFSD6, INPP1, HIBCH, C2H2orf88, MSTN, PMS1, ORMDL1, OSGEPL1, ANKAR, ASNSD1, SLC40A1, WDR75,COL5A2, MIR2917, COL3A1, DIRC1,GULP1* |
|  | 14 | BovineHD1400007190 | Hapmap40958-BTA-34312 | *SDCBP, NSMAF, TOX, TRNAC-GCA, CA8, RAB2A, CHD7* |
| **MUP** | 5 | 5_74951342 | 5_75130860 | *CACNG2* |
| **PON** | 5 | BovineHD0500021258 | 5_75130860 | *MYH9, TXN2, FOXRED2, EIF3D, TRNAE-UUC, CACNG2* |

Assembly Bos_taurus_UMD_3.1
